# Lactate and lactylation in sepsis-associated acute kidney injury: clinical evidence from the MIMIC-IV database and mechanistic insights

**DOI:** 10.3389/fmed.2025.1708145

**Published:** 2025-11-14

**Authors:** Sujing Zhang, Mengyuan Luo, Zhijie Lu, Qiqing Shi

**Affiliations:** 1 Department of Anesthesiology, Minhang Hospital, Fudan University, Shanghai, China; 2 Department of Anesthesiology and Perioperative Medicine, Shanghai Fourth People’s Hospital, School of Medicine, Tongji University, Shanghai, China

**Keywords:** sepsis, acute kidney injury, lactate, lactate clearance, protein lactylation, MIMIC-IV, CRRT

## Abstract

**Background:**

Sepsis-associated acute kidney injury (SA-AKI) is a common and severe complication of sepsis, yet early predictors remain limited. Lactate, beyond being a marker of tissue hypoperfusion, may act as a signaling molecule through protein lactylation. This study aimed to investigate the association between lactate levels and SA-AKI risk using the MIMIC-IV database and to explore the potential mechanistic role of lactylation in septic mice.

**Methods:**

Adult sepsis patients were identified from the MIMIC-IV (v3.1) database. Patients were stratified by lactate tertiles within 24 h of ICU admission, and lactate clearance (LC) was assessed as a dynamic indicator. The primary outcome was SA-AKI, and secondary outcomes included CRRT use and 28-day mortality. Kaplan–Meier analysis, Cox regression, and restricted cubic spline (RCS) models were performed. In parallel, a murine cecal ligation and puncture (CLP) model was used to evaluate tissue-specific protein lactylation by Western blot, along with serum lactate and hematological parameters.

**Results:**

A total of 11,431 patients were included. Higher lactate levels were associated with increased disease severity, higher incidence and severity of SA-AKI, greater use of CRRT, and elevated 28-day ICU and in-hospital mortality. In Cox regression, lactate as both a continuous and categorical variable was independently associated with SA-AKI risk. RCS analysis revealed nonlinear dose–response relationships, with sharply increased risk of SA-AKI above 5.7 mmol/L. In sensitivity analyses (*n* = 6,593), higher LC was associated with lower risks of SA-AKI, CRRT use, and mortality. Cox regression confirmed LC as an independent protective factor, while RCS suggested a downward but non-significant trend. In the CLP mouse model, protein lactylation was markedly elevated in the kidney but not in the heart, liver, or spleen, accompanied by higher serum lactate.

**Conclusion:**

Elevated lactate levels are independently associated with increased risk of SA-AKI in sepsis patients, whereas higher lactate clearance is linked to improved renal outcomes. Moreover, kidney-specific upregulation of protein lactylation in septic mice suggests a possible molecular link between lactate metabolism and renal vulnerability. These findings highlight lactate and lactylation as both prognostic markers and potential mechanistic contributors in SA-AKI.

## 1 Introduction

Sepsis is a life-threatening syndrome caused by a dysregulated host response to infection, with persistently high global incidence and mortality rates (1–3). Acute kidney injury (AKI) is one of the most common complications of sepsis, occurring in approximately 40–50% of cases, and is closely associated with prolonged hospitalization and increased mortality risk (4–6). Early identification of high-risk patients and timely intervention are crucial for improving outcomes.

Lactate is a widely used clinical biomarker for assessing tissue perfusion and metabolic status. Elevated lactate levels often indicate tissue hypoxia, mitochondrial dysfunction, and dysregulated inflammatory responses. In recent years, it has been proposed that lactate is not merely a bystander of metabolic disturbance but may also act as a signaling molecule directly involved in pathological processes (7, 8). Lactate clearance (LC), as a dynamic indicator, has shown greater prognostic value than single lactate measurements in some studies (9, 10). In addition, the discovery of protein lactylation has provided a new perspective for understanding the role of lactate in organ dysfunction. Lactylation is a novel epigenetic modification that can regulate transcriptional activity, inflammatory responses, and energy metabolism, thereby influencing the function of multiple organs, including the kidney. Basic research has suggested that lactylation may play a key role in amplifying inflammation and promoting kidney injury in sepsis, but clinical evidence remains limited (11, 12).

At present, the clinical evidence regarding the association between lactate levels, lactate clearance, and sepsis-associated AKI (SA-AKI) is still insufficient. Previous studies have mainly focused on the relationship between lactate and mortality, while systematic evaluations of kidney outcomes are scarce. Meanwhile, the role of lactylation in SA-AKI has only recently gained attention, with most available evidence derived from animal experiments, and integrated clinical–mechanistic studies are still lacking.

Therefore, in this study, we utilized the large-scale critical care database MIMIC-IV to systematically evaluate the relationship between early lactate levels, lactate clearance, and the risk of SA-AKI in patients with sepsis. Furthermore, we established a cecal ligation and puncture (CLP) mouse model to investigate the potential role of protein lactylation in kidney injury. We hypothesized that elevated lactate and insufficient lactate clearance are significantly associated with an increased risk of SA-AKI, and that lactylation may represent an important molecular pathway mediating this relationship, thereby providing new insights for clinical risk assessment and mechanistic exploration.

## 2 Materials and methods

### 2.1 Study population

This study utilized data from the MIMIC-IV v3.1 database (2008–2022), which was jointly developed by the Massachusetts Institute of Technology (MIT) and Beth Israel Deaconess Medical Center (BIDMC). The database has been de-identified in compliance with the Health Insurance Portability and Accountability Act (HIPAA). The corresponding author of this study obtained access to the database (Record ID: 71467776); therefore, no additional ethical approval was required.

**Figure 1:**
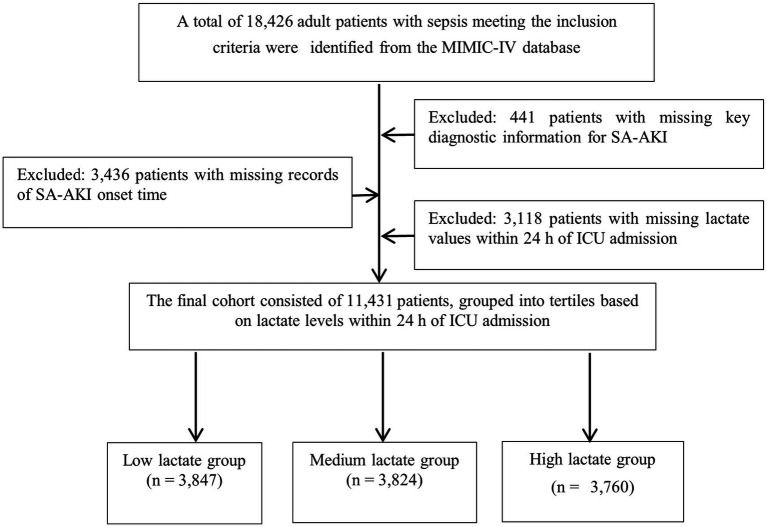
Flowchart of patient selection. A total of 11,431 patients with sepsis were included after applying inclusion and exclusion criteria, and were stratified into tertiles according to lactate levels within the first 24 h of ICU admission.

The inclusion criteria were as follows: (1) age ≥18 years; (2) first ICU admission; (3) diagnosis of sepsis according to the Sepsis-3 definition; (4) availability of lactate measurement within 24 h after ICU admission; and (5) at least two serum creatinine or urine output records for the assessment of AKI. Exclusion criteria included: (1) pre-existing AKI or receipt of CRRT prior to ICU admission; (2) missing key variables; (3) repeated hospitalizations (only the first ICU stay was included); and (4) ICU length of stay <24 h. A total of 11,431 patients were finally included in the analysis (Figure 1).

### 2.2 Variables extraction and processing

Data extraction was performed using DecisionChain software (version 1.0). The primary exposure variable was the highest lactate level within 24 h after ICU admission. Patients were divided into low-, medium-, and high-lactate groups according to tertiles of the highest lactate value within 24 h after ICU admission. Likewise, lactate clearance (LC) was stratified into tertiles based on the percentage change between lactate levels at 24 h and 48 h following ICU admission. This tertile-based grouping approach was chosen to ensure a balanced sample distribution and to facilitate trend analysis across increasing lactate or clearance levels. Covariates included demographic information, severity scores, comorbidities, laboratory variables, treatment measures, and outcomes. Variables with missing rates >20% (e.g., height, albumin) were excluded, while those with ≤20% missing values were imputed using multiple imputation.

### 2.3 Study outcomes

The primary outcome was the occurrence of sepsis-associated acute kidney injury (SA-AKI). Baseline serum creatinine values were obtained directly from the MIMIC-IV database. Patients lacking sufficient renal function data were excluded. SA-AKI was diagnosed according to the Kidney Disease: Improving Global Outcomes (KDIGO) 2012 criteria, defined as any of the following: an increase in serum creatinine by ≥0.3 mg/dL within 48 h; an increase to ≥1.5 times the baseline within 7 days; or urine output <0.5 mL/kg/h for at least 6 h. The secondary outcome was the initiation of continuous renal replacement therapy (CRRT).

### 2.4 Animal experiments

Mice were anesthetized with sevoflurane (2–3% in oxygen, delivered via an induction chamber at a flow rate of 2 L/min) prior to surgery. A cecal ligation and puncture (CLP) procedure was performed to establish a murine model of sepsis. Mice were randomly assigned to a sham control group or a CLP group (*n* = 4 per group). At 24 h after surgery, mice were euthanized under deep anesthesia with sevoflurane (5% in oxygen, 2 L/min, maintained for >3 min until complete loss of reflexes) before sample collection. Blood samples were collected via enucleation for routine blood counts (white blood cell counts) and for serum lactate measurement by ELISA. The heart, liver, spleen, and kidneys were subsequently harvested, washed in PBS, and stored at −80 °C. Protein samples were subjected to SDS-PAGE and transferred onto NC membranes. Immediately after transfer, membranes were stained with Ponceau S solution to confirm equal protein loading and uniform transfer. After blocking with 5% non-fat milk for 1 h at room temperature, membranes were incubated overnight at 4 °C with a pan anti-lactylation antibody (PTM BIO, Hangzhou, China; Cat. No. PTM-1401RM, Lot: RM101402; dilution 1:1,000), followed by incubation with HRP-conjugated secondary antibodies for 1 h at room temperature. Membranes were then washed with TBST and visualized using HRP-based chemiluminescence. Band intensities were quantified using ImageJ software and normalized to total protein (Ponceau S staining). Given the limited sample size (*n* = 4 per group), this animal experiment was conducted as a preliminary exploratory study aimed at identifying the organ-specific pattern of protein lactylation during sepsis.

All animal experiments were conducted in accordance with the Guide for the Care and Use of Laboratory Animals and were approved by the Ethics Committee for Laboratory Animal Use at Fudan University (Approval No. 2025-MHYY-031).

### 2.5 Statistical analysis

Continuous variables were analyzed according to their distribution. Normally distributed data were expressed as mean ± standard deviation (SD) and compared using the independent-samples *t*-test or one-way analysis of variance (ANOVA). Non-normally distributed data were expressed as median (interquartile range, IQR) and compared using the Mann–Whitney *U* test or Kruskal–Wallis test. Categorical variables were presented as counts and percentages, and compared using the chi-square test or Fisher’s exact test.

Survival analysis was performed using Kaplan–Meier (KM) methods to plot cumulative incidence curves, and group differences were assessed by the log-rank test.

Cox proportional hazards regression models were applied to evaluate the association between lactate levels and the risk of SA-AKI. Univariate Cox regression was first conducted to screen potential confounders. Subsequently, three multivariable models were constructed: Model 1, unadjusted; Model 2, adjusted for age, sex, and ethnicity; and Model 3, further adjusted for comorbidities including hypertension, chronic kidney disease, diabetes mellitus, liver cirrhosis, heart failure, and chronic obstructive pulmonary disease. Results were reported as hazard ratios (HRs) with 95% confidence intervals (CIs).

Restricted cubic spline (RCS) models were used to explore the dose–response relationship between lactate levels and SA-AKI risk, testing both linear and nonlinear associations (based on three or four knots).

To verify the robustness of the findings, sensitivity analyses were performed by substituting lactate clearance (LC) for static lactate levels. LC was calculated as:

LC (%) = (Lac₍_24 h_₎ – Lac₍_48 h_₎)/Lac₍_24 h_₎ × 100%. RCS models were further applied to assess the relationship between LC and the risk of SA-AKI.

All statistical analyses were conducted using DecisionChain software (version 1.0; Hangzhou Yuantong Information Technology Co., Ltd., Hangzhou, China). A two-tailed *p*-value <0.05 was considered statistically significant.

## 3 Results

### 3.1 Baseline characteristics stratified by lactate tertiles

A total of 11,431 patients with sepsis were included in this study, of whom 6,728 (58.9%) were male, with a median age of 68 years (IQR: 57–79 years). Based on lactate levels within the first 24 h of ICU admission, patients were stratified into tertiles: the low lactate group [1.20 (1.00–1.40) mmol/L, *n* = 3,847], the medium lactate group [1.92 (1.74–2.15) mmol/L, *n* = 3,824], and the high lactate group [3.44 (2.82–4.61) mmol/L, *n* = 3,760] (Table 1). Compared with the low lactate group, patients in the high lactate group were younger, with a higher proportion of males and non-White individuals. As lactate levels increased, platelet counts decreased, while white blood cell counts, blood glucose, and serum creatinine levels progressively increased, accompanied by a decline in arterial pH. Both SOFA and SAPS II scores increased stepwise across the tertiles. Furthermore, the incidence and severity of AKI were significantly higher in the high lactate group, along with increased use of vasoactive agents (VP), mechanical ventilation, and CRRT. The 28-day in-hospital and ICU mortality rates were also markedly elevated in the high lactate group (all *p* < 0.01). Overall, higher lactate levels were closely associated with greater disease severity, an increased risk of SA-AKI, and worse clinical outcomes.

**Table 1:** Baseline characteristics of 11,431 sepsis patients stratified by lactate tertiles.

Variables	Overall (*N* = 11,431)	Low lactate group (*N* = 3,847)	Medium lactate group (*N* = 3,824)	High lactate group (*N* = 3,760)	*p*-value
LA, median (IQR)	1.90 (1.40–2.80)	1.20 (1.00–1.40)	1.92 (1.74–2.15)	3.44 (2.82–4.61)	
Demographics					
Age, median (IQR)	68 (57–79)	68 (57–79)	69 (58–79)	67 (55–78)	<0.001
Gender, *n* (%)					0.003
Female	4,703 (41.14)	1,662 (43.20)	1,506 (39.38)	1,535 (40.82)	
Male	6,728 (58.86)	2,185 (56.80)	2,318 (60.62)	2,225 (59.18)	
Weight, median (IQR)	82.45 (69.00–98.30)	82.40 (68.50–99.10)	82.84 (69.30–98.05)	82.20 (69.35–98.00)	0.854
Ethnicity, *n* (%)					<0.001
White	7,107 (62.17)	2,490 (64.73)	2,436 (63.70)	2,181 (58.01)	
Black	715 (6.25)	230 (5.98)	232 (6.07)	253 (6.73)	
Other	3,609 (31.57)	1,127 (29.30)	1,156 (30.23)	1,326 (35.27)	
Laboratory tests					
Plt, median (IQR)	173.50 (122.50–240.00)	196.50 (143.00–266.25)	173.67 (125.50–237.25)	151.55 (104.29–212.58)	<0.001
WBC, median (IQR)	12.60 (9.24–16.86)	11.65 (8.60–15.30)	12.80 (9.70–16.93)	13.69 (9.50–18.53)	<0.001
Glu, median (IQR)	134.50 (112.00–170.50)	126.00 (106.83–153.60)	133.59 (113.84–165.50)	148.71 (119.75–190.50)	<0.001
pH, median (IQR)	7.37 (7.32–7.41)	7.38 (7.33–7.42)	7.38 (7.33–7.41)	7.34 (7.29–7.39)	<0.001
Scr, median (IQR)	1.13 (0.80–1.84)	1.03 (0.73–1.70)	1.10 (0.80–1.70)	1.30 (0.93–2.00)	<0.001
Scales					
SOFA, median (IQR)	7.00 (4.00–9.00)	6.00 (4.00–8.00)	6.00 (4.00–9.00)	8.00 (6.00–11.00)	<0.001
SAPS II, median (IQR)	42.00 (33.00–52.00)	39.00 (31.00–49.00)	41.00 (33.00–50.00)	47.00 (37.00–58.00)	<0.001
Diagnosis					
AKI, *n* (%)					<0.001
No	5,640 (49.34)	2,123 (55.19)	2,016 (52.72)	1,501 (39.92)	
Yes	5,791 (50.66)	1,724 (44.81)	1,808 (47.28)	2,259 (60.08)	
AKI stage, *n* (%)					<0.001
1	1,931 (16.89)	685 (17.81)	701 (18.33)	545 (14.49)	
2	5,072 (44.37)	1,863 (48.43)	1,797 (46.99)	1,412 (37.55)	
3	4,428 (38.74)	1,299 (33.77)	1,326 (34.68)	1,803 (47.95)	
Interventions					
VP, *n* (%)					<0.001
No	2,711 (23.72)	1,238 (32.18)	900 (23.54)	573 (15.24)	
Yes	8,720 (76.28)	2,609 (67.82)	2,924 (76.46)	3,187 (84.76)	
Ventilation, *n* (%)					<0.001
No	3,727 (32.60)	1,381 (35.90)	1,331 (34.81)	1,015 (26.99)	
Yes	7,704 (67.40)	2,466 (64.10)	2,493 (65.19)	2,745 (73.01)	
CRRT, *n* (%)					<0.001
No	10,068 (88.08)	3,518 (91.45)	3,489 (91.24)	3,061 (81.41)	
Yes	1,363 (11.92)	329 (8.55)	335 (8.76)	699 (18.59)	
Outcome					
In-hospital 28 day mortality, *n* (%)					<0.001
No	8,856 (77.47)	3,155 (82.01)	3,077 (80.47)	2,624 (69.79)	
Yes	2,575 (22.53)	692 (17.99)	747 (19.53)	1,136 (30.21)	
In-ICU 28 day mortality, *n* (%)					<0.001
No	8,772 (76.74)	3,127 (81.28)	3,055 (79.89)	2,590 (68.88)	
Yes	2,659 (23.26)	720 (18.72)	769 (20.11)	1,170 (31.12)	

Data are presented as median (interquartile range, IQR) or *n* (%). LA, lactate; Plt, platelet; WBC, white blood cell; Glu, glucose; pH, blood gas pH value; SCr, serum creatinine; SOFA, Sequential Organ Failure Assessment; SAPS II, Simplified Acute Physiology Score II; AKI, acute kidney injury; CRRT, continuous renal replacement therapy; VP, vasoactive agents.

### 3.2 Baseline characteristics stratified by SA-AKI

Table 2 shows the baseline characteristics of patients stratified by the occurrence of SA-AKI. Patients in the SA-AKI group had significantly higher lactate levels compared with those without SA-AKI, and were older, with a higher proportion of males, greater body weight, and a higher prevalence of non-White ethnicity. Regarding comorbidities, the SA-AKI group had higher rates of liver cirrhosis (LCi), chronic kidney disease (CKD), type 2 diabetes mellitus (T2DM), heart failure (HF), and chronic obstructive pulmonary disease (COPD), whereas the prevalence of hypertension (HTN) was relatively lower.

**Table 2:** Baseline characteristics of patients with and without SA-AKI.

Variables	Overall (*N* = 11,431)	No SA-AKI group (*N* = 5,640)	SA-AKI group (*N* = 5,791)	*p*-value
Lac, median (IQR)	1.90 (1.40–2.80)	1.80 (1.30–2.50)	2.05 (1.45–3.20)	<0.001
Demographics				
Age, median (IQR)	68 (57–79)	67.00 (56–78)	69.00 (57–80)	<0.001
Gender, *n* (%)				<0.001
Female	4,703 (41.14)	2,385 (42.29)	2,318 (40.03)	
Male	6,728 (58.86)	3,255 (57.71)	3,473 (59.97)	
Weight, median (IQR)	82.45 (69.00–98.30)	80.78 (68.19–96.13)	84.00 (70.00–100.20)	<0.001
Ethnicity, *n* (%)				<0.001
White	7,107 (62.17)	3,638 (64.50)	3,469 (59.90)	
Black	715 (6.25)	277 (4.91)	438 (7.56)	
Other	3,609 (31.57)	1,725 (30.59)	1,884 (32.53)	
Comorbidities (%)				
HTN, *n* (%)				<0.001
No	6,850 (59.92)	3,035 (53.81)	3,815 (65.88)	
Yes	4,581 (40.08)	2,605 (46.19)	1,976 (34.12)	
LCi, *n* (%)				<0.001
No	10,181 (89.06)	5,292 (93.83)	4,889 (84.42)	
Yes	1,250 (10.94)	348 (6.17)	902 (15.58)	
CKD, *n* (%)				<0.001
No	9,107 (79.67)	5,028 (89.15)	4,079 (70.44)	
Yes	2,324 (20.33)	612 (10.85)	1,712 (29.56)	
CA, *n* (%)				0.174
No	9,739 (85.20)	4,831 (85.66)	4,908 (84.75)	
Yes	1,692 (14.80)	809 (14.34)	883 (15.25)	
T1DM, *n* (%)				
No	11,273 (98.62)	5,569 (98.74)	5,704 (98.50)	0.265
Yes	158 (1.38)	71 (1.26)	87 (1.50)	
T2DM, *n* (%)				
No	8,040 (70.34)	4,225 (74.91)	3,815 (65.88)	<0.001
Yes	3,391 (29.66)	1,415 (25.09)	1,976 (34.12)	
HF, *n* (%)				
No	7,775 (68.02)	4,197 (74.41)	3,578 (61.79)	<0.001
Yes	3,656 (31.98)	1,443 (25.59)	2,213 (38.21)	
IHD, *n* (%)				0.543
No	7,015 (61.37)	3,477 (61.65)	3,538 (61.09)	
Yes	4,416 (38.63)	2,163 (38.35)	2,253 (38.91)	
COPD, *n* (%)				<0.001
No	9,536 (83.42)	4,795 (85.02)	4,741 (81.87)	
Yes	1,895 (16.58)	845 (14.98)	1,050 (18.13)	
Scales				
SOFA, median (IQR)	7.00 (4.00–9.00)	5.00 (4.00–8.00)	8.00 (5.00–11.00)	<0.001
SAPS II, median (IQR)	42.00 (33.00–52.00)	37.00 (30.00–46.00)	47.00 (38.00–57.00)	<0.001
Outcomes				
Hospital stay (day), median (IQR)	12.06 (7.21–20.70)	10.20 (6.50–16.97)	14.13 (8.32–23.95)	<0.001
ICU stay (day), median (IQR)	5.25 (3.20–9.87)	4.54 (2.99–8.32)	6.14 (3.64–11.49)	<0.001
In-hospital 28 day mortality, *n* (%)				<0.001
No	8,856 (77.47)	4,850 (85.99)	4,006 (69.18)	
Yes	2,575 (22.53)	790 (14.01)	1,785 (30.82)	
In-ICU 28 day mortality, *n* (%)				<0.001
No	8,772 (76.74)	4,833 (85.69)	3,939 (68.02)	
Yes	2,659 (23.26)	807 (14.31)	1,852 (31.98)	

Data are presented as median (interquartile range, IQR) or *n* (%). SA-AKI, sepsis-associated acute kidney injury; HTN, hypertension; LC, liver cirrhosis; CKD, chronic kidney disease; CA, cancer; T1DM, type 1 diabetes mellitus; T2DM, type 2 diabetes mellitus; HF, heart failure; IHD, ischemic heart disease; COPD, chronic obstructive pulmonary disease; SOFA, Sequential Organ Failure Assessment; SAPS II, Simplified Acute Physiology Score II.

In addition, SOFA and SAPS II scores were significantly higher in the SA-AKI group. Both ICU and hospital lengths of stay were prolonged, and 28-day in-hospital and ICU mortality rates were markedly higher in patients with SA-AKI than in those without (all *p* < 0.001). These findings suggest that patients with SA-AKI typically present with higher lactate levels, greater disease burden, and worse clinical outcomes.

### 3.3 Lactate levels and renal outcomes: Kaplan–Meier analysis

**Figure 2:**
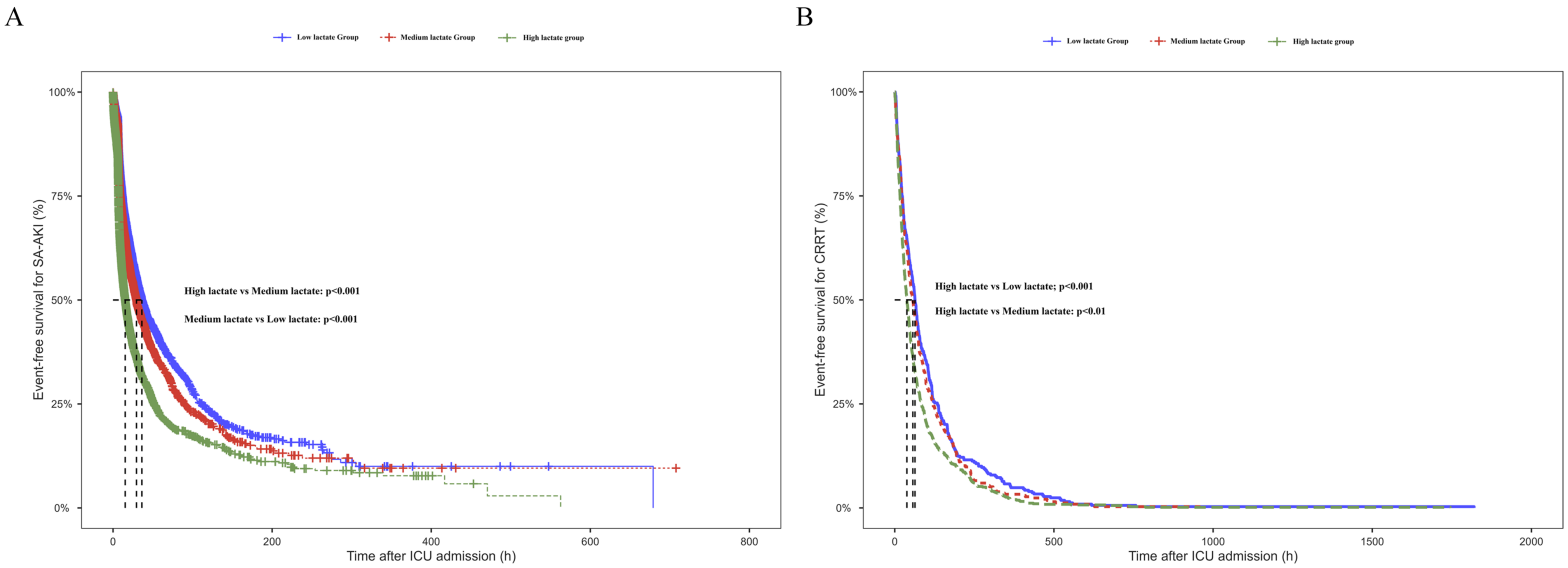
Kaplan–Meier analysis of renal outcomes stratified by lactate tertiles. **(A)** Cumulative event-free survival for SA-AKI according to lactate tertiles. Patients in the high lactate group developed SA-AKI significantly earlier than those in the low and medium groups (log-rank *p* < 0.001). **(B)** Cumulative event-free survival for CRRT according to lactate tertiles. The risk of CRRT differed significantly among groups (log-rank *p* < 0.001), with patients in the high lactate group showing earlier and more frequent use of CRRT.

Figure 2A shows the cumulative event-free survival curves for SA-AKI stratified by lactate tertiles. The risk of SA-AKI differed significantly among the groups (log-rank *p* < 0.001). Higher lactate levels were associated with earlier onset of SA-AKI and a faster decline in event-free survival, with the high lactate group showing the steepest decrease.

Figure 2B presents the cumulative incidence curves of CRRT across the lactate groups. The incidence of CRRT differed significantly among the three groups (log-rank *p* < 0.001). No significant difference was observed between the low and medium lactate groups (*p* = 0.236), whereas patients in the high lactate group required CRRT earlier and more frequently compared with both the low and medium groups (all *p* < 0.01). These findings indicate that higher lactate levels were not only associated with an increased risk of SA-AKI but also with more severe kidney injury requiring renal replacement therapy.

### 3.4 Lactate levels and the risk of SA-AKI: multivariable Cox regression analysis

In multivariable Cox regression analysis, lactate (Lac) as a continuous variable remained significantly associated with an increased risk of SA-AKI: Model 1, HR = 1.15 (95% CI: 1.135–1.162, *p* < 0.001); Model 2, HR = 1.16 (95% CI: 1.148–1.171, *p* < 0.001); and Model 3, HR = 1.14 (95% CI: 1.129–1.152, *p* < 0.001). When LC was analyzed categorically, both the medium and high clearance groups showed a reduced risk of SA-AKI compared with the low group. The association was significant for both groups in model 1, attenuated for the high group in model 2, but remained robust in the fully adjusted model 3 (medium: HR = 0.858, 95% CI: 0.798–0.922; high: HR = 0.895, 95% CI: 0.830–0.965; both *p* < 0.001; Table 3). These findings indicate that elevated lactate levels, whether analyzed as a continuous or categorical variable, were significantly associated with an increased risk of SA-AKI, supporting lactate as an independent predictor in sepsis patients.

**Table 3:** Association between lactate levels and the risk of SA-AKI in different Cox regression models.

Exposure (lactate)	Model 1 HR (95% CI)	Model 2 HR (95% CI)	Model 3 HR (95%) CI
Continuous variable (per 1 mmol/L increase)	1.15 (1.135–1.162)^***^	1.16 (1.148–1.171)^***^	1.14 (1.129–1.152)^***^
Categorical variable (tertiles)			
Low lactate group (reference)	1	1	1
Medium lactate group	1.174 (1.099–1.254)^***^	1.173 (1.098–1.254)^***^	1.101 (1.03–1.177)^**^
High lactate group	1.731 (1.625–1.843)^***^	1.783 (1.674–1.899)^***^	1.281 (1.198–1.369)^***^

HR, hazard ratio; CI, confidence interval; SA-AKI, sepsis-associated acute kidney injury. Model 1: unadjusted. Model 2: adjusted for age, sex, and ethnicity. Model 3: further adjusted for comorbidities (hypertension, chronic kidney disease, diabetes mellitus, liver cirrhosis, heart failure, and chronic obstructive pulmonary disease). For categorical analysis, the low lactate group was used as the reference. ^**^p < 0.01 and ^***^p < 0.001.

### 3.5 Dose–response relationship between lactate levels and renal outcomes: restricted cubic spline analysis

**Figure 3:**
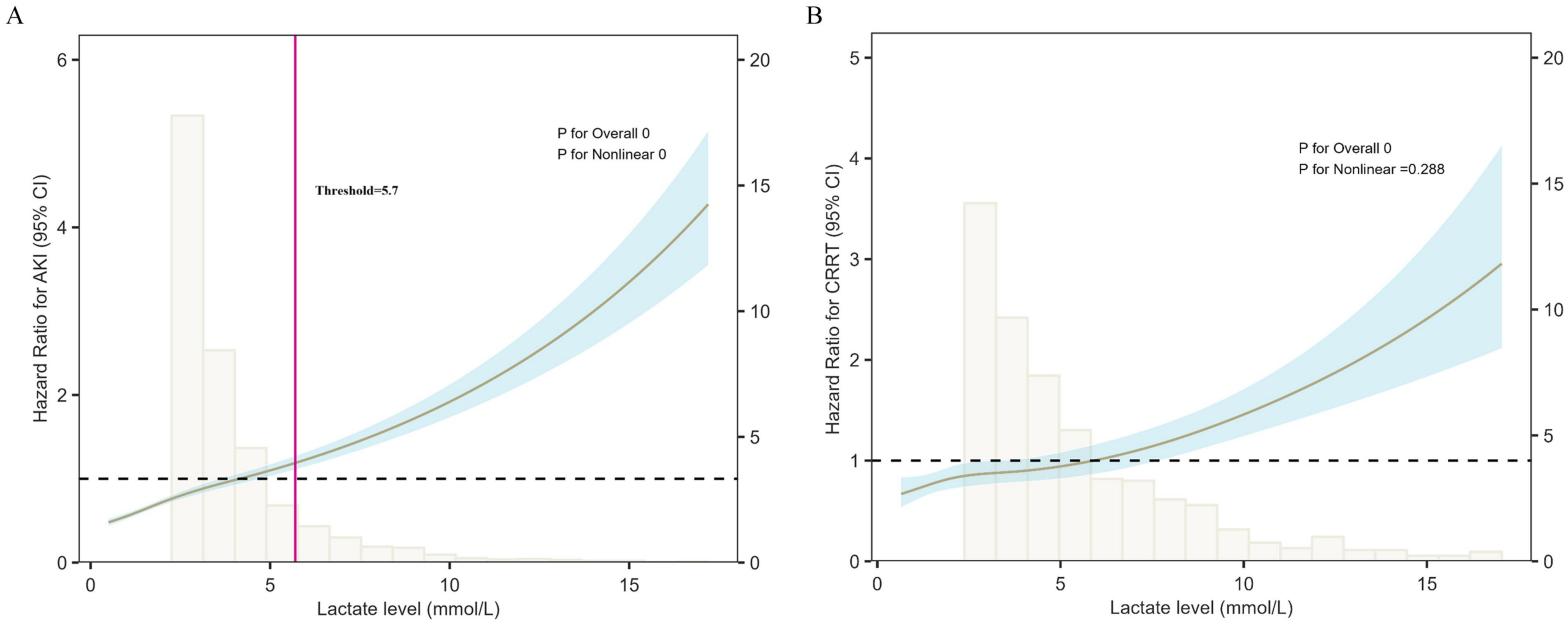
Restricted cubic spline (RCS) analysis of lactate levels and renal outcomes. **(A)** Lactate levels showed a nonlinear association with SA-AKI risk (overall *p* < 0.001, nonlinearity *p* < 0.001). The risk increased slowly at lower levels but rose sharply when lactate exceeded ~5.7 mmol/L, marking an inflection point that may represent a threshold for metabolic decompensation and sharply elevated SA-AKI risk. **(B)** Lactate levels were significantly associated with the risk of CRRT (*p* < 0.001), with an approximately linear increase across the range. Shaded areas represent 95% confidence intervals.

To further explore the relationship between lactate levels and renal outcomes, a restricted cubic spline (RCS) model was applied. Figure 3A illustrates the association between lactate levels and the risk of SA-AKI. A significant nonlinear dose–response pattern was observed (overall *p* < 0.001, nonlinearity p < 0.001). At lower lactate levels, the risk of SA-AKI increased only modestly, whereas beyond approximately 5.7 mmol/L, the risk rose steeply, indicating a substantially higher likelihood of SA-AKI in patients with elevated lactate. The inflection point at approximately 5.7 mmol/L may represent a threshold beyond which lactate accumulation reflects metabolic decompensation and a sharp increase in the risk of SA-AKI.

Figure 3B presents the association between lactate levels and the risk of CRRT based on the RCS model. A significant overall association was observed (*p* < 0.001), while no evidence of nonlinearity was detected (*p* = 0.288). The risk of CRRT increased almost linearly with rising lactate levels, indicating that patients with higher lactate concentrations were more likely to require renal replacement therapy.

### 3.6 Sensitivity analysis: lactate clearance and renal outcomes

To verify the robustness of the findings, lactate clearance (LC) was further evaluated as a dynamic indicator. A total of 6,593 patients were included, and LC was calculated using lactate values at 24 h and 48 h after ICU admission. Patients were stratified into low (LC-L), medium (LC-M), and high (LC-H) clearance groups according to tertiles (Table 4). As LC increased, the incidence of SA-AKI decreased progressively; similarly, the use of CRRT and both 28-day in-hospital and ICU mortality rates declined across groups (all *p* < 0.001).

**Table 4:** Baseline characteristics and outcomes stratified by lactate clearance tertiles.

Variables	Overall (*N* = 6,593)	LC-L group (*N* = 2,219)	LC-M group (*N* = 2,191)	LC-H group (*N* = 2,183)	*p*-value
LC, median (IQR)	0.24 (0.00–0.46)	−0.12 (−0.35–0.00)	0.25 (0.17–0.32)	0.54 (0.46–0.63)	<0.001
AKI, *n* (%)					<0.001
No	2,601 (39.45)	783 (35.29)	874 (39.89)	944 (43.24)	
Yes	3,992 (60.55)	1,436 (64.71)	1,317 (60.11)	1,239 (56.76)	
CRRT, *n* (%)					<0.001
No	5,432 (82.39)	1,717 (77.38)	1,839 (83.93)	1,876 (85.94)	
Yes	1,161 (17.61)	502 (22.62)	352 (16.07)	307 (14.06)	
In-hospital 28 day mortality, *n* (%)					<0.001
No	4,745 (71.97)	1,418 (63.90)	1,618 (73.85)	1,709 (78.29)	
Yes	1,848 (28.03)	801 (36.10)	573 (26.15)	474 (21.71)	
In-ICU 28 day mortality, *n* (%)					<0.001
No	4,682 (71.01)	1,392 (62.73)	1,594 (72.75)	1,696 (77.69)	
Yes	1,911 (28.99)	827 (37.27)	597 (27.25)	487 (22.31)	

Data are presented as median (interquartile range, IQR) or *n* (%). LC, lactate clearance; AKI, acute kidney injury; CRRT, continuous renal replacement therapy.

**Figure 4:**
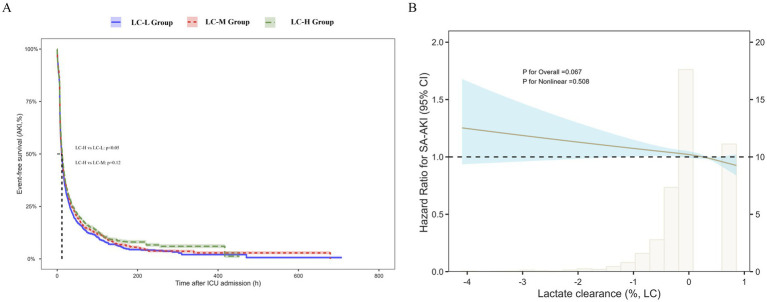
Sensitivity analysis of lactate clearance (LC) and renal outcomes. **(A)** Kaplan–Meier survival curves stratified by LC tertiles. Overall survival did not differ significantly among groups (log-rank *p* = 0.084), but patients in the high LC group showed significantly better survival compared with the low LC group (*p* < 0.05). **(B)** Restricted cubic spline (RCS) analysis of LC and the risk of SA-AKI. A downward trend in SA-AKI risk was observed with higher LC, although the association did not reach statistical significance (*p* for overall = 0.067; *p* for nonlinear = 0.508). Shaded areas represent 95% confidence intervals.

Kaplan–Meier analysis showed no significant overall difference in survival among the three groups (log-rank *p* = 0.084, Figure 4A). However, pairwise comparison revealed that survival was significantly higher in the LC-H group compared with the LC-L group (*p* < 0.05), indicating that higher lactate clearance was associated with better prognosis.

In multivariable Cox regression analysis, LC as a continuous variable was inversely associated with the risk of SA-AKI (Model 1 HR = 0.939, 95% CI 0.887–0.994; Model 2 HR = 0.933, 95% CI 0.881–0.989; Model 3 HR = 0.940, 95% CI 0.887–0.996; all *p* < 0.001). When analyzed as tertiles, patients in the high clearance group generally had a lower risk of SA-AKI compared with those in the low clearance group (Model 1 HR = 0.919, 95% CI 0.852–0.992; Model 2 HR = 0.994, 95% CI 0.876–1.017; Model 3 HR = 0.895, 95% CI 0.830–0.965; Table 5).

**Table 5:** Association between lactate clearance and the risk of SA-AKI in Cox regression models.

Exposure (lactate clearance, LC)	Model 1 HR (95% CI)	Model 2 HR (95% CI)	Model 3 HR (95%) CI
Continuous variable	0.939 (0.887–0.994)^***^	0.933 (0.881–0.989)^***^	0.940 (0.887–0.996)^***^
Categorical variable (tertiles)			
Low lactate group (reference)	1	1	1
Medium lactate group	0.977 (0.906–1.053)	0.858 (0.794–0.927)^***^	0.858 (0.794–0.927)^***^
High lactate group	0.919 (0.852–0.992)^*^	0.994 (0.876–1.017)	0.895 (0.83–0.965)^**^

HR, hazard ratio; CI, confidence interval; SA-AKI, sepsis-associated acute kidney injury. Model 1: unadjusted. Model 2: adjusted for age, sex, and ethnicity. Model 3: further adjusted for comorbidities (hypertension, chronic kidney disease, diabetes mellitus, liver cirrhosis, heart failure, and chronic obstructive pulmonary disease). For categorical analysis, the LC-L group was used as the reference. ^*^p < 0.05, ^**^p < 0.01, and ^***^p < 0.001.

RCS analysis further demonstrated a downward trend between LC and the risk of SA-AKI (Figure 4B). Although the association did not reach statistical significance (*p* for overall = 0.067; *p* for nonlinear = 0.508), the trend still suggested that higher lactate clearance may be linked to a reduced risk of SA-AKI.

### 3.7 Specific elevation of protein lactylation in the kidneys of septic mice

Recent studies have demonstrated that lactate is not only a metabolic byproduct of tissue hypoperfusion and metabolic disturbances but also acts as a signaling molecule through protein lactylation, thereby regulating inflammation and gene expression. In the context of SA-AKI, protein lactylation may serve as a key molecular mechanism linking metabolic dysregulation to organ injury.

**Figure 5:**
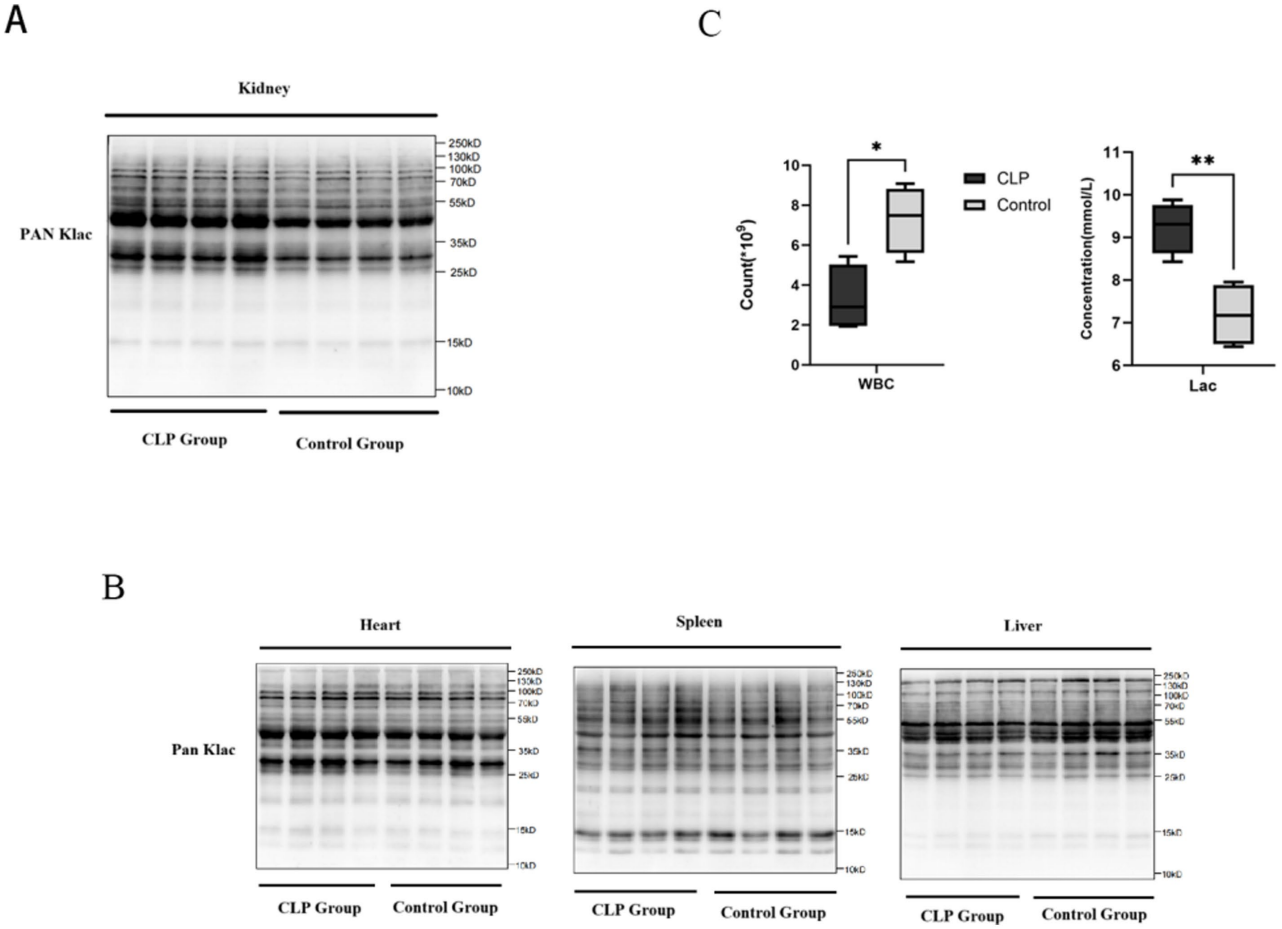
Protein lactylation and serum parameters in the CLP mouse model. **(A)** Western blot analysis of kidney tissues showing that protein lactylation levels were markedly elevated in CLP mice compared with sham controls. **(B)** Western blot analysis of heart, liver, and spleen tissues revealed no significant differences in protein lactylation between CLP and sham groups. **(C)** ELISA results demonstrated significantly higher serum lactate levels in CLP mice (*p* < 0.01), while routine blood tests showed a significant reduction in leukocyte counts compared with the sham group (*p* < 0.05).

In this study, we established a murine sepsis model using the CLP procedure and collected kidney, heart, liver, and spleen tissues 24 h after surgery for Western blot analysis. Ponceau S staining confirmed comparable total protein loading across all tissues and groups (Supplementary Figure 1A). After normalization to total protein, quantitative analysis revealed that global protein lactylation was significantly increased in the kidneys of CLP mice compared with sham-operated controls (Figure 5A), whereas no significant differences were observed in the heart, liver, or spleen (Figure 5B; Supplementary Figure 1B). In addition, ELISA results indicated markedly elevated serum lactate levels in the CLP group, while routine blood tests revealed significantly reduced leukocyte counts compared with the sham group (Figure 5C).

Taken together, these findings suggest that during the early stage of sepsis, the kidney is the first organ to exhibit a significant increase in protein lactylation. This indicates that lactylation may represent a potential mechanism by which elevated lactate mediates renal vulnerability. Moreover, it highlights that lactate is not only a marker of metabolic disturbance but may also participate in inflammatory and immune dysregulation through lactylation, thereby contributing to the development and progression of SA-AKI.

## 4 Discussion

In this study, we systematically evaluated the association between lactate levels, lactate clearance, and sepsis-associated acute kidney injury (SA-AKI) in more than 11,000 patients with sepsis from the MIMIC-IV database, and further explored potential mechanisms using a CLP mouse model. Our findings demonstrated that elevated lactate levels within the first 24 h after ICU admission were independently associated with an increased risk of SA-AKI, following a nonlinear dose–response pattern, with a sharp rise in risk observed when lactate concentrations exceeded approximately 5.7 mmol/L. In contrast, higher lactate clearance exhibited a protective effect, being associated not only with a lower risk of SA-AKI but also with reduced CRRT utilization and improved overall outcomes.

Lactate has long been recognized as a classical indicator of tissue hypoperfusion and metabolic distress, and it has been widely used to assess sepsis severity and predict mortality. Numerous clinical studies have consistently demonstrated that elevated lactate levels are strongly associated with increased mortality, leading to the incorporation of lactate monitoring into the Surviving Sepsis Campaign (SSC) guidelines (13–15). However, most previous investigations have focused primarily on overall mortality or multiple organ dysfunction, whereas acute kidney injury (AKI) has rarely been examined as a distinct outcome. Leveraging a large-scale critical care database, the present study confirmed an independent association between elevated lactate levels and the development of SA-AKI, and further identified a nonlinear dose–response pattern. Notably, an RCS-derived inflection point around 5.7 mmol/L may signify a critical metabolic transition during sepsis, at which lactate production exceeds clearance and tissue hypoxia becomes evident. Clinically, patients whose lactate levels surpass this threshold appear to be at substantially greater risk for SA-AKI, underscoring the importance of early renal-protective strategies.

In recent years, increasing attention has been given to the clinical significance of lactate clearance (LC) as a dynamic indicator. Previous studies have shown that lactate clearance reflects tissue perfusion and the effectiveness of resuscitation more accurately than single-point lactate measurements, and it is closely associated with mortality risk. For example, Nguyen et al. (16) reported that a lactate clearance ≥10% was significantly associated with improved 28-day survival. Similarly, Jones et al. (10), in a randomized controlled trial, demonstrated that targeting lactate clearance as a resuscitation goal could reduce mortality. The present study further supports these findings, showing that patients in the intermediate and high clearance groups had a significantly lower risk of SA-AKI, along with reduced CRRT utilization and overall mortality. Integrating baseline lactate levels and clearance rates into routine ICU workflows may enhance early recognition and management of patients at risk for SA-AKI. Baseline lactate provides an initial assessment of metabolic stress at admission, whereas serial measurements within the first 24–48 h dynamically reflect tissue perfusion and the effectiveness of resuscitation. Incorporating lactate clearance targets into standardized sepsis bundles or electronic decision-support systems could enable real-time risk stratification and timely implementation of renal-protective interventions. By combining absolute lactate values with their clearance trends, clinicians can obtain a more comprehensive and actionable understanding of metabolic status. Although the prognostic role of lactate is well recognized, our findings extend its clinical relevance by specifically linking lactate dynamics to SA-AKI risk and by proposing a practical framework for integrating these indicators into renal-protective management.

Mechanistic experiments further revealed that the kidney was the earliest organ to exhibit a significant increase in protein lactylation in the CLP model, whereas no comparable changes were detected in the heart, liver, or spleen, suggesting that lactylation may represent a potential molecular link underlying lactate-mediated renal vulnerability. The novelty of this work lies in the integration of large-scale clinical data with animal experiments, thereby validating the close relationship between lactate and SA-AKI from both an epidemiological and mechanistic perspective.

The discovery of lactylation has provided a novel perspective for understanding lactate-mediated organ injury. Previous studies have shown that histone H3K18 lactylation (H3K18la) promotes the transcription of inflammation-related genes and is associated with SOFA and APACHE II scores as well as mortality (11, 17, 18), whereas non-histone lactylation events (such as those involving Fis1, HMGB1, and Ezrin) can exacerbate mitochondrial dysfunction, inflammatory responses, and immune dysregulation (18–20). In the present study, we observed in the CLP mouse model that the kidney was the earliest organ to exhibit a marked increase in protein lactylation, suggesting potential organ specificity and a heightened susceptibility of the kidney to lactate metabolic disturbances. However, these findings from the animal experiment are exploratory and should be interpreted with caution, as the small sample size limits definitive mechanistic inference.

These findings open new avenues for clinical translation. Lactylation-related molecules such as H3K18la may emerge as novel adjunctive indicators to improve predictive accuracy. From a therapeutic perspective, targeting lactate metabolism and lactylation-associated pathways may hold potential, including inhibition of lactate dehydrogenase (LDH), blockade of monocarboxylate transporters (MCTs), or modulation of lactylation-regulating enzymes such as p300/CBP and SIRT3 (21–24). However, challenges remain, including limited specificity, potential drug-related adverse effects, and technical limitations in detection. Looking ahead, integrating multi-omics approaches (transcriptomics, proteomics, and metabolomics) with machine learning algorithms to construct comprehensive predictive models may accelerate the transition of lactate and lactylation from prognostic biomarkers to therapeutic targets (25–27).

Of course, this study has several limitations. First, as a retrospective analysis, although a large sample size was included and multiple confounding factors were adjusted for, the ability to establish causality remains limited and cannot fully substitute for prospective studies. Second, because the MIMIC-IV database originates from a single tertiary academic center, potential institutional and regional biases cannot be excluded. The lack of external validation in independent or multicenter cohorts limits the generalizability and external applicability of our results. Therefore, future studies with prospective, multicenter validation are essential to confirm the robustness and reproducibility of these findings across diverse populations and clinical settings. Third, the animal experiments were conducted with a relatively small sample size and short observation period. Although our data demonstrated a strong association between elevated lactate levels, enhanced protein lactylation, and the development of SA-AKI, these findings mainly reflect correlation rather than causation. We did not evaluate the expression of key lactylation-regulating enzymes such as LDHA, SIRT1, or KAT2B, nor perform interventional experiments using lactate transporter inhibitors or lactylation-blocking peptides. Future studies should adopt systematic experimental approaches to investigate these mechanistic pathways and determine whether modulation of lactylation can mitigate renal injury in sepsis.

## 5 Conclusion

Based on large-scale clinical data from the MIMIC-IV database combined with findings from a CLP mouse model, this study systematically elucidated the clinical association and potential mechanistic implications between lactate and sepsis-associated acute kidney injury (SA-AKI). Elevated lactate levels were significantly associated with an increased risk of SA-AKI, whereas higher lactate clearance was closely linked to improved renal outcomes. At the mechanistic level, the kidney was identified as the earliest organ to exhibit a marked increase in protein lactylation, suggesting that lactylation may represent a key molecular link underlying lactate metabolism-driven renal vulnerability. Taken together, this study not only establishes the clinical value of lactate and lactate clearance as risk assessment indicators for SA-AKI but also provides new evidence supporting the role of lactylation in its pathogenesis, thereby laying a theoretical foundation for future predictive and interventional strategies.

## Data Availability

The raw data supporting the conclusions of this article will be made available by the authors, without undue reservation.
