# Supplementary material for: Lactate and lactylation in sepsis-associated acute kidney injury: clinical evidence from the MIMIC-IV database and mechanistic insights
Source: Front Med (Lausanne). 2025 Nov 14;12:1708145. doi: 10.3389/fmed.2025.1708145 (PMC12660216; doi:10.3389/fmed.2025.1708145)
Supplement: Supplementary file 1 [file Data_Sheet_1.pdf]

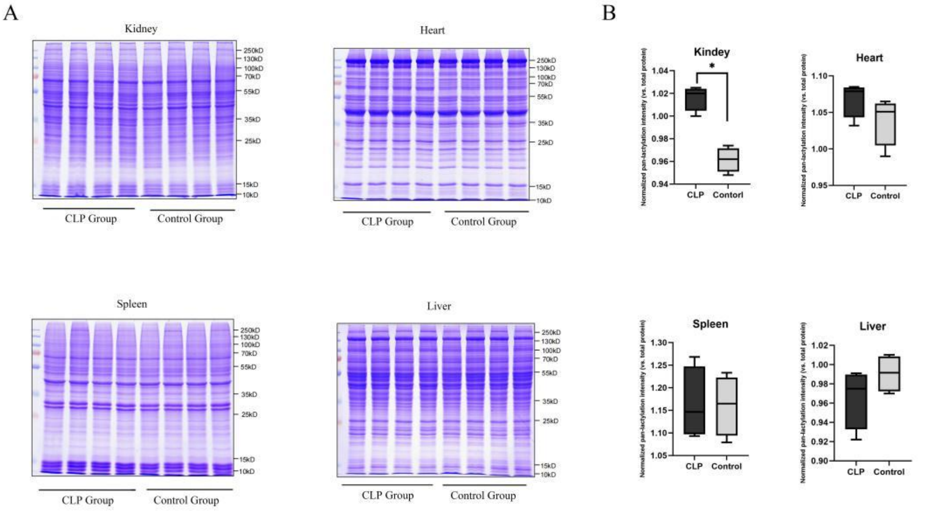

Supplementary Figure 1. Organ-specific analysis of total protein and pan-lactylation levels in septic and control mice. (A) Ponceau S staining of total protein following Western blot transfer in kidney, heart, spleen, and liver tissues from cecal ligation and puncture (CLP) and sham-operated mice. No significant difference in total protein loading was observed among tissues or between groups. (B) Quantification of normalized pan-lactylation intensity (vs. total protein). Compared with the control group, only the kidney exhibited a significant increase in protein lactylation after CLP-induced sepsis ($P < 0.05$). Data are expressed as mean ± SD (n = 4 per group), analyzed using unpaired Student's *t*-test.
